# Process Parameter Optimization of Extrusion-Based 3D Metal Printing Utilizing PW–LDPE–SA Binder System

**DOI:** 10.3390/ma10030305

**Published:** 2017-03-16

**Authors:** Luquan Ren, Xueli Zhou, Zhengyi Song, Che Zhao, Qingping Liu, Jingze Xue, Xiujuan Li

**Affiliations:** Key Laboratory of Bionic Engineering (Ministry of Education), Jilin University, Changchun 130022, China; lqren@jlu.edu.cn (L.R.); zysong16@mails.jlu.edu.cn (Z.S.); zhaoche14@mails.jlu.edu.cn (C.Z.); xuejz15@mails.jlu.edu.cn (J.X.); xiujuanli@jlu.edu.cn (X.L.)

**Keywords:** extrusion-based printing process, metal additive manufacturing, copper powders, orthogonal test

## Abstract

Recently, with a broadening range of available materials and alteration of feeding processes, several extrusion-based 3D printing processes for metal materials have been developed. An emerging process is applicable for the fabrication of metal parts into electronics and composites. In this paper, some critical parameters of extrusion-based 3D printing processes were optimized by a series of experiments with a melting extrusion printer. The raw materials were copper powder and a thermoplastic organic binder system and the system included paraffin wax, low density polyethylene, and stearic acid (PW–LDPE–SA). The homogeneity and rheological behaviour of the raw materials, the strength of the green samples, and the hardness of the sintered samples were investigated. Moreover, the printing and sintering parameters were optimized with an orthogonal design method. The influence factors in regard to the ultimate tensile strength of the green samples can be described as follows: infill degree > raster angle > layer thickness. As for the sintering process, the major factor on hardness is sintering temperature, followed by holding time and heating rate. The highest hardness of the sintered samples was very close to the average hardness of commercially pure copper material. Generally, the extrusion-based printing process for producing metal materials is a promising strategy because it has some advantages over traditional approaches for cost, efficiency, and simplicity.

## 1. Introduction

With the rapid development of additive manufacturing (AM), metal AM has attracted more and more attention. At present, laser melting and high energy electron beam melting are the most widely-used AM methods. However, there are still many disadvantages which seriously limit the development of metal AM, especially reflected in the shortage of available materials.

Recently, with a broadening range of available materials and alteration of feeding processes, several extrusion-based printing techniques for metal materials have been developed. The extrusion-based printing technique combined with the fluid-dispensing system and automated robotic system is used for extrusion and printing. Raw metallic materials are generally prepared by metal powder paste and binder. Then, the paste is fed into the fluid-dispensing system driven by aerodynamic pressure or mechanical transmission (piston or screw-driven). The curing process includes cooling solidification and cross-linked solidification, etc. Some researchers have classified the extrusion-based printing technique into different types according to the forming mechanism and the binder that is used. Fused deposition modeling (FDM) has become the most widely used AM method because of its simplicity and inexpensiveness. Nevertheless, only the thermoplastic filaments can be used in the FDM process—such as acrylonitrile butadiene styrene (ABS) [1,2] and polylactic acid (PLA) [3]. Guohua W et al. [4] described a hard tooling fabrication technique named fused deposition of metals (FDMet). FDMet is a modified FDM process, in which three-dimensional (3D) objects are built using metal filament fed into a heated extruder, but the fabrication of the fused filament has stringent requirements in the raw materials property and temperature control. Uwe Sheithauer et al. [5,6] developed a new additive manufacturing technique—thermoplastic 3D printing (3DTP)—by combining the process of FDM and robocasting. The high-load feedstock with thermoplastic binder systems is extruded through a heated nozzle. 3DTP could be used for the preparation of ceramic (alumina/zirconia [5]), metal (copper [7]), and composites (steel–zirconia [6]). Still, it takes a long time to remove the binder system comprised of paraffin, beeswax, and dispersing agent. Otherwise, the sample will be distorted. Another newly developed process for metal materials is termed as 3D gel-printing (3DGP) [8]. The 3DGP process has been reported to be used for fabrication of part of 316L steel utilizing the methaerylate-2-hydroxy ethyl (HEMA) gelation system and stainless steel powder. The procedure is as follows: premixed solution preparation, slurry preparation, initiator addition, and printing. However, a series of crosslinking reactions are hardly controlled. Meanwhile, the dispersion of initiator, curing time, and speed cannot be regulated accurately. Direct write printing (DWP) [9,10,11,12,13] can eject inks through an extrusion needle under an appropriate pressure from the nitrogen pump. The extrusion needle can move under the computer instructions and drive the extrusion delivery system. The DWP process can be used for producing different materials such as zirconium dioxide and 3D Silk/Hydroxyapatite etc. The deficiency of DWP is that it cannot be used to print metal parts. Nano-particle jet printing has been developed as a mature commercial 3D printing technology, especially for applications in electronics and phonotics. Nanoparticles (e.g., Au and Ag) are important functional materials due to their high conductivity, chemical stability, and resistance to surface oxidation [14,15,16], but the preparation process of the ink is complex, and the requirement of the powder material is high.

Moreover, the successful additive manufacturing process depends upon the proper selection of process parameters. Determination of the optimum process conditions is a challenging task for the producer. Therefore, the process variables need to be researched and optimized precisely. However, the research on this aspect is not very mature at the present stage. Most of the research has been focused on improving the mechanical properties and quality of thermoplastic polymer parts fabricated by traditional FDM [17]. More quantitative investigations should be carried out to explore the parameter optimization of the extrusion-based 3D metal printing technique.

**Figure 1:**
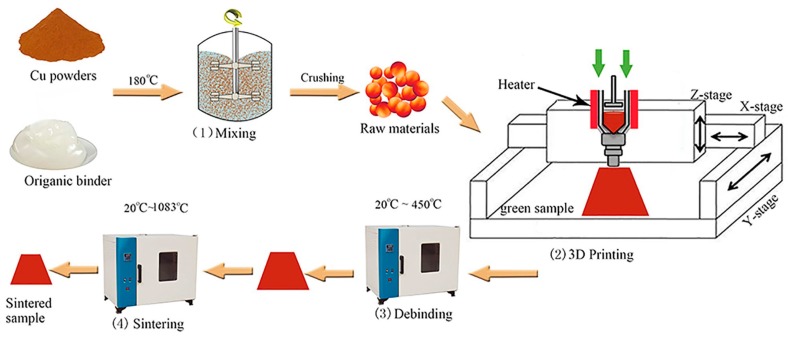
Schematic of the technological process.

In this research, the experimental study is about the influence factor of the mechanical properties of the printed parts and sintered samples. The raw materials were composed of copper powder and the thermoplastic organic binder system. This thermoplastic organic binder system is relatively mature in powder injection molding (PIM) [18,19,20,21], which includes paraffin wax, low density polyethylene, and stearic acid (PW–LDPE–SA). The slurry-like molten feedstock can be extruded via a thin nozzle. Figure 1 shows the schematic of the technological process in this paper. The process comprises the following four steps: (1) mixing of the metal powder and organic binder to prepare raw materials; (2) green part 3D printing; (3) green part debinding to remove the binder from the component; (4) sintering to consolidate the debinding component. This manufacturing method combines the traditional shape-making capability of the extrusion-based printing process and the material flexibility of powder metallurgy.

## 2. Experimental Section

### 2.1. Materials

The following polymer materials were used in the study: paraffin wax (PW, China National Petroleum Corporation, Beijing, China, product mark 58#), low density polyethylene (LDPE, Formosa Plastics Corporation, Zhejiang, China, product mark 3470, density 0.924 g/cm^3^), stearic acid (SA, Tianjin Guangfu Fine Chemical Research Institute, China, molecular weight 284.48). Copper powders were (purity >99.5%, grain size 200 mesh) also provided by Tianjin Guangfu Fine Chemical Research Institute.

### 2.2. Raw Materials Preparation

The composition of the raw materials was listed in Table 1, which is one of the representative ratios suggested in previous work [18,19,20,21]. First, the paraffin wax, low-density polyethylene, and stearic acid were mixed at 180 °C as an organic binder until the mixture was uniform. Then, Cu powders were added into the organic binder and the whole system was thoroughly mixed. Finally, the materials were crushed into particles smaller than 3 mm.

### 2.3. 3D Printing Process

To print the granular raw material and construct 3D structures, piston-type extrusion equipment was self-developed. The raw materials were housed in heated syringes, which were attached to a nozzle [6,22]. Equipment was used to pressurize the barrel and control the flow rate of raw materials’ fusion. A schematic diagram of the printing equipment is shown in Figure 1(2).

### 2.4. Post-Process

Post-process was divided into two stages: debinding and sintering. The printed samples were debinded using thermal debinding. Post-process was performed in a vacuum atmosphere furnace (KJ-A1400-12LY, Zhengzhou Kejia Electric Furnace Co. Ltd., Zhengzhou, China) by heating up to 500 °C at 2 °C/min, holding time for 150 min, then continuing to raise the temperature for sintering.

### 2.5. Orthogonal Experiment Design

Orthogonal experimental design is a study of the multifactorial nature and standard of design methods through the part of the test to find out the optimal level combination. Compared with the complete test, the partial test needs fewer experiments. It is possible to use the corresponding range analysis method, variance analysis method, and regression analysis method to analyze the test results, thus many valuable and reliable conclusions may be obtained. The optimum technological conditions and order of experiment factors on the target index were determined by orthogonal testing and the range analysis method.

Firstly, in the printing process, the orthogonal experiment was designed with the ultimate tensile strength of the printing parts as the index—with layer thickness, raster angle, and infill degree as factors. Table 2 shows the variables and levels selected for study. In orthogonal test design, the table of L9 (34) was adopted. To investigate the relationships between printing factors and mechanical properties, nine samples with different printing parameters were created using the raw material. Optimal parameter combination was obtained by experimental results analyses and optimization of printing parameters. 

Secondly, sintering is the most important process in the preparation process and can consolidate the sample after debinding effectively. The sintering approach and scheme are closely correlated with the properties of the final products. In the sintering process, the experimental optimization design method also was used to optimize the heat treatment craft parameters on hardness. The optimization factors include the heating rate, highest temperature, and holding time. The evaluated parameters of the sintering process were summarized in Table 3.

### 2.6. Characterization

The particle-size distribution was tested by a laser particle analyzer (BT-9300ST, Dandong Baxter instrument Co., Ltd, Dandong, China). The appearance of the Cu particle and energy dispersive X-ray analyses (EDX) on the surface of the sintered specimen were characterized by a scanning electric microscope (SEM, ZEISS EVO 18, Carl Zeiss AG, Cambridge, UK). The density and porosity of the sample were measured using the Archimedes immersion method in water. Rheological experiments of the raw materials were carried out at 160 °C on an Anton Paar 302 rheometer (Anton Paar (Shanghai) Trading Co., Ltd., Shanghai, China) with the protection of N_2_ and plate–plate geometry (diameter of 25 mm, steel) being used. The apparent viscosity (η) is acquired as a function of shear rate (10^−1^–10^3^) in a logarithmically ascending series. Frequency sweeps at a shear strain of 0.1% from 10^−1^ to 10^2^ rad/s performed to record the storage modulus (G′) and loss modulus (G″) as a function of frequency. The tensile strength of the printed and sintered samples was measured using an electronic universal testing machine (DDL100, Changchun research institute for mechanical science Co., Ltd., Changchun, China) at a tension speed of 1 mm/min, as recommended in ASTM D638-2003 and ASTM E8, respectively. For each printed/sintered sample production condition, four tensile tests were performed. The hardness of the sintered samples was determined by the Vickers hardness tester (HV-1000, Shanghai Jvjing Precision Instrument Manufacturing Co., Ltd., Shanghai, China). For each sintered sample production condition, three hardness tests were performed. In this study, thermogravimetric analysis (TGA) experiments were performed using LINSEIS STA PT 1600 (Linseis, Bavaria zell cloth, Germany) to assess the weight change, which is reported as a function of temperature. The tests were conducted over a temperature range of 25 °C to 700 °C in a vacuum, and the heating rates were 2, 5, and 10 °C/min. The metallographic microstructure of the sintered specimen was characterized by a metallurgical microscope (ZEISS AXIO, Carl Zeiss AG, Cambridge, UK). XRD patterns were recorded in an X-ray diffractometer (D/Max2500, Rigaku Corporation, The Woodlands, TX, USA) with Cu Kα radiation, a voltage of 50 kV, and a current of 200 mA. The 2θ region analyzed was 3°–100° with continuous scanning.

## 3. Results and Discussion

### 3.1. Materials and Rheological Behaviour

**Figure 2:**
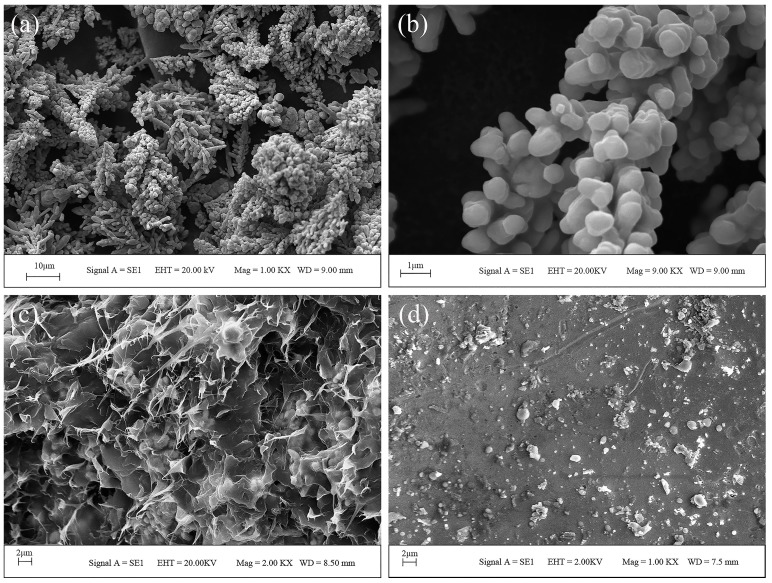
SEM image of the Cu powder with the magnifying power of 1 kx (**a**); the Cu powder with the magnifying power of 9 kx (**b**); cross section of the green body (**c**); surface of the sintered part with heating rate (3 °C/min), highest temperature (1083 °C), holding time (3 h) (**d**).

Figure 2a shows the SEM image of the Cu powder and the magnification morphology is shown in Figure 2b. As shown in Figure 2a,b, the Cu powders were irregularly shaped. Table 4 shows the characteristics of copper powder obtained by a laser particle size analyzer; the results show that the mean particle size is 35.57 μm.

Since the homogeneity of raw materials is the key to achieving successful extrusion through the nozzle, we also observed the distribution of the powder and binder in the raw materials. As shown in Figure 2c, copper particles were homogeneously dispersed and completely surrounded in the thermoplastic binder, which was composed of paraffin wax, low-density polyethylene, and stearic acid. Besides, the density of the raw materials was measured by the Archimedes method. The average density of the raw materials was 2.695 g/cm^3^. The standard deviation and the maximum difference between the measured densities of the raw materials were 0.0178 and 0.160 g/cm^3^, respectively. In summary, the raw materials are mixed evenly.

**Figure 3:**
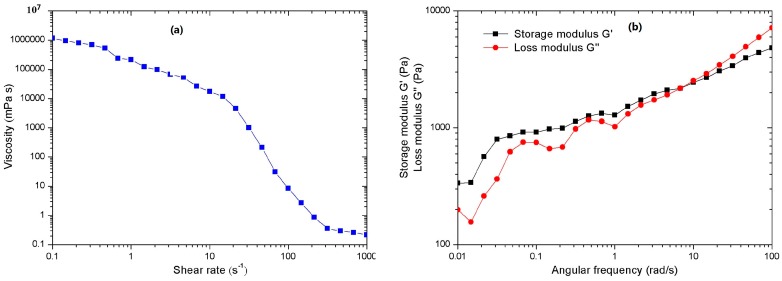
Rheological behaviour of the raw materials at 160 °C: (**a**) the viscosity as a function of shear rate; (**b**) the storage modulus (G′) and loss modulus (G″) as a function of frequency.

The rheological behaviours have a direct impact on the smooth extrusion of the raw materials. Figure 3a shows the rheological property of raw materials and the apparent viscosity as a function of shear rate. Raw materials exhibit obvious shear thinning behaviour. This effect ensures the printability of the raw materials at low pressures, so that shear force is easily imparted to the fused raw materials inside the nozzle. Besides, the storage modulus (G′) and loss modulus (G″) of raw materials in the whole extrusion process were also studied. We find that G′ was greater than G″ at low angular frequency as shown in Figure 3b, which indicates liquid-like behaviour. However, at high angular frequency above (10 rad/s~100 rad/s), G′ was less than G″, this means that the materials are endowed with solid-like behaviour [23]. This behaviour is important for the shape retention of the printing process at this temperature, as the materials can rapidly recover their solid-like behaviour after exiting the nozzle. 

### 3.2. Printing Process Optimization

**Figure 4:**
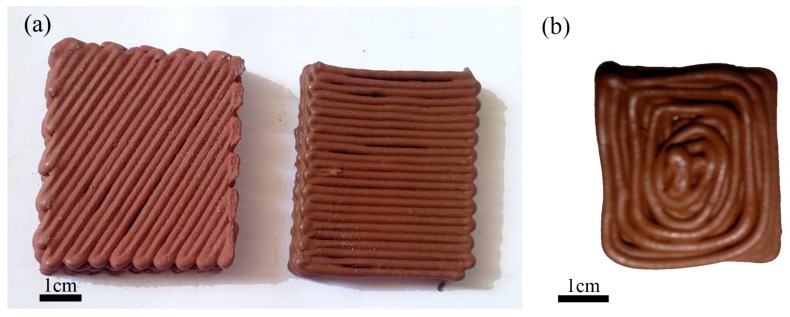
Green samples printed by this method with: (**a**) appropriate printing conditions; (**b**) inappropriate printing conditions.

To print the prepared raw materials, printing conditions were optimized. These conditions are very important for printing prepared materials; unlike traditional extrusion 3D printing, for example FDM, the printed material is granular. In order to obtain a fine 3D structure, the printing conditions list in Table 5 was used. Figure 4 shows optical photographs of green samples printed under various operating conditions. Figure 4a shows that the fine 3D structure is obtained by the optimum printing conditions. An example without optimization of printing conditions is shown in Figure 4b.

**Figure 5:**
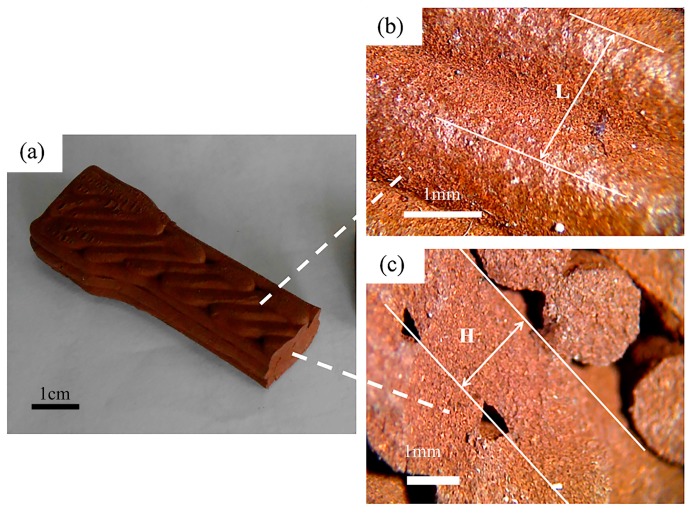
Green sample printed by this method: (**a**) overview; (**b**) the top surface of the green sample; (**c**) cross section of the green sample.

**Figure 6:**
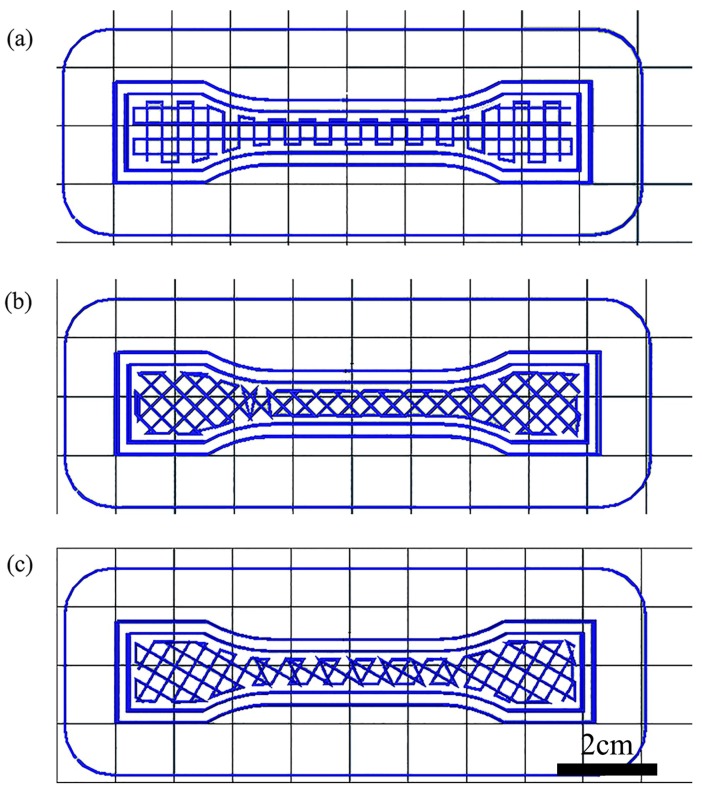
Types of raster angle used in 3D printing of the tensile test specimens: (**a**) 0°/90°; (**b**) ±45°; (**c**) 60°/−30°.

In addition to obtaining the complete shape, the strength of the printing samples also needs to be investigated, because changes in the shape caused by insufficient strength and the mechanical failure of the green body would occur [24]. An example was printed as shown in Figure 5a. The distance of printed lines (L) and layer thickness (H) can be observed in Figure 5b,c. Figure 5c shows the size of the voids, which directly affects the mechanical properties of the print samples. It can be influenced by many factors, such as layer thickness, raster angle, infill degree, build orientations, etc. Layer thickness is the thickness of the layer deposited by the nozzle. The raster angle is the direction of the raster with respect to the loading direction of stress (Figure 6). Infill degree directly reflects the distance between two adjacent deposited filaments in the same layer. The work described herein investigated the influence of layer thickness, raster angle and infill degree on the mechanical properties of green samples built by the self-developed 3D printing system [25]. We properly designed an orthogonal experiment in which there were three factors, each with three levels of orthogonal experiments, making nine experiments in total. The effect of different factors can be evaluated by the range (R) calculated from the results of the orthogonal experiment. As presented in Table 6, by test data analysis, the influence degree of different factors and the optimum formulation of the ultimate tensile strength are obtained.

**Figure 7:**
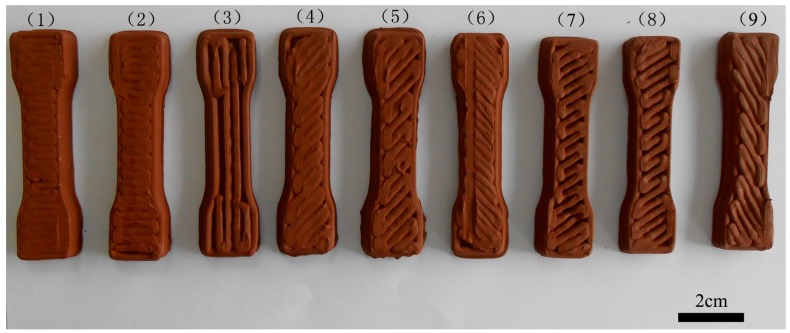
Green samples with different printing parameters corresponding to the data of Table 6.

Based on the results obtained from the orthogonal design, the range analysis is adopted to determine the sensitivity of the factors to the orthogonal design. The *R_J_* was defined as:

$$R_{J}=\max\left(\bar{y_{ji}}\right)-\min\left(\bar{y_{ji}}\right)$$

where “$\max\left(\bar{y_{ji}}\right)$” represents the maximum value of $\bar{y_{ji}}$ and “$\min\left(\bar{y_{ji}}\right)$” the minimum value of $\bar{y_{ji}}$. The *j* stands for the factor in Table 2 and Table 3, *i* stands for the level of the selected factor, from 1 to 3. Figure 7 shows the samples printed at different parameters corresponding to the data in Table 6. From Table 6 and Figure 2c, we can see that the mechanical properties of the printed samples are not solely controlled by the organic binder of the raw material but also significantly influenced by a directional dependent production process. High infill degree is known to produce finished AM parts that withstand higher tensile stress. A number of researchers have drawn these conclusions in their studies similar to our hypothesis [26]. The raster angle can affect the internal structure of the finished product. There is always an angle perpendicular to the immediate preceding layer between two adjacent layers. Angle variation also influences tensile strength, although not very significantly. The influence of different factors on the ultimate tensile strength of the sample can be described as follows: infill degree > raster angle > layer thickness. The influence induced by infill degree on the experiments is extremely remarkable. The orthogonal test showed that the optimum combination of printing parameters was +45°/−45° with 2.0 mm layer thickness and 80% infill degree. 

### 3.3. Warpage Deformation of the Green Samples

**Figure 8:**
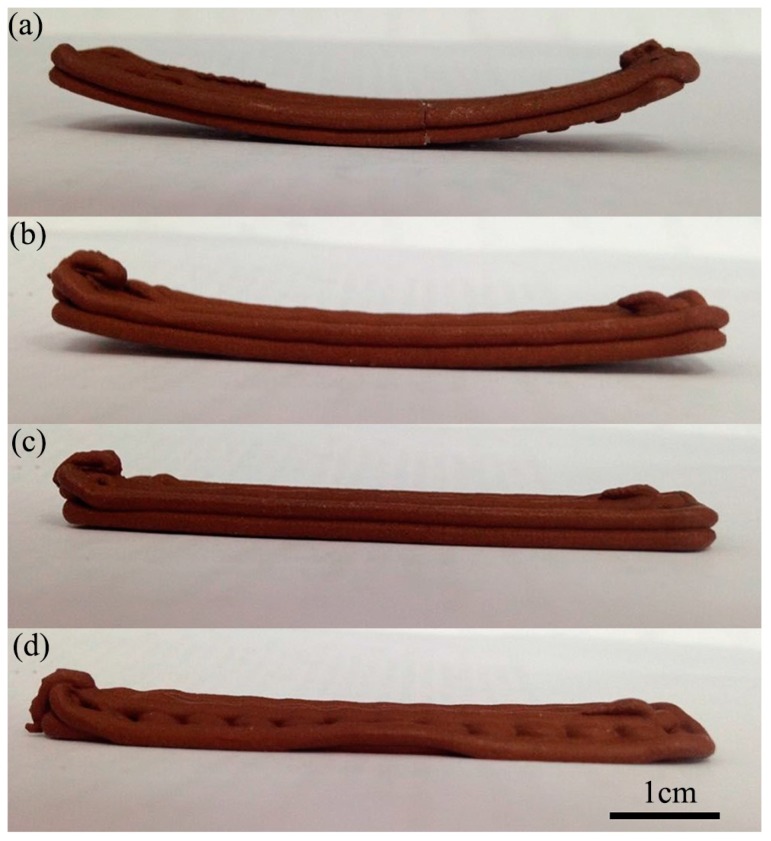
Green samples printed by a novel additive manufacturing method with: (**a**) the temperature of the substrate 30 °C; (**b**) the temperature of the substrate 50 °C; (**c**) the temperature of the substrate 70 °C; (**d**) the temperature of the substrate 90 °C.

In addition to the above operating conditions, a few details are worth highlighting. Improving the shaping precision of the prototype and reducing the warp deformation are also emphasized in this field. The high-temperature raw material extruded out of the nozzle will deposit on the substrate following the scheduled road path. When cooling from the melted temperature *T*_m_ to the glass-transition temperature *T*_g_, the deposited thermoplastic raw material will shrink. In spite of the contraction, the inner stress of the deposited raw material is not accumulated; this is because when the cooling temperature ranges from *T*_m_ to *T*_g_, the deposited raw material can acquire a larger deformation with less force, and the capacity to resist outside force is small. The inner stress is mainly produced in the course of the cooling process, when the temperature changes from the glass-transition temperature *T*_g_ to the room temperature *T*_e_ [27]. We improve this defect by raising the temperature of the substrate. From Figure 8, we know that the degree of warp deformation of green parts would vary with the temperature of the substrate. When the temperature of the substrate is too high (70–90 °C), the printed lines would collapse. Finally, the printed layers would collapse and the part cannot be formed. On the other hand, when the temperature of the substrate is low (~30 °C), green parts would bend. Obviously, the printed sample would lose dimensional accuracy. So, we will set the temperature of the substrate at 50 °C; at this temperature, the printing sample will not produce deflection.

### 3.4. Debinding

**Figure 9:**
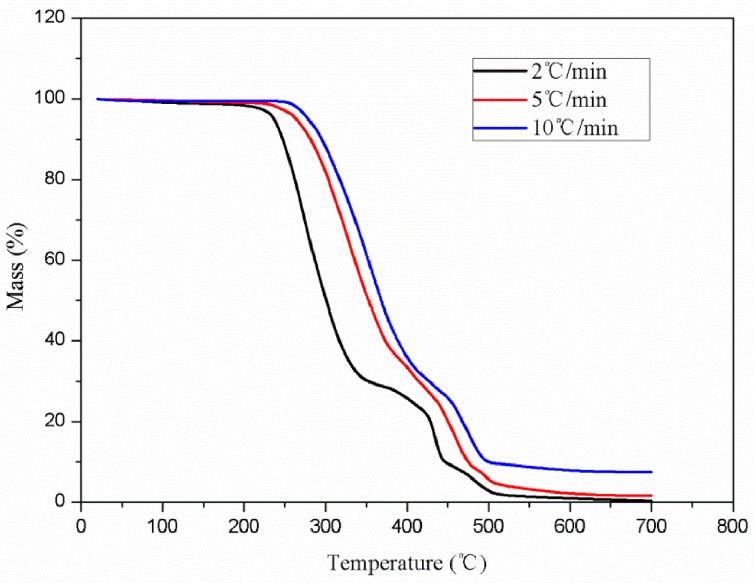
Thermogravimetric Analysis (TGA) curves of the organic binder with heating rates of 2, 5, and 10 °C/min.

Thermal debinding was used in the process. When the temperature reaches the melting point of the binder, the binder starts to melt. When the temperature continues to rise and reaches the thermal decomposition temperature of the binder, the binder will be thermally decomposed, thereby producing a small molecule compound of gas. Thermogravimetric Analysis (TGA) was performed to determine the appropriate debinding temperature. The TG curves of the organic binder with heating rates of 2, 5, and 10 °C/min are shown in Figure 9. The thermal decomposition of the specimen heated at 2 °C/min is thorough. Obviously, the organic binder began to decompose at about 250 °C. When the temperature is in the range of 300 °C to 500 °C, the organic binder decomposed violently. Hence, the debinding temperature of the green sample was determined to conduct at 500 °C for 150 min in a vacuum atmosphere furnace such that the organic binder can decompose completely.

### 3.5. Sintering Process Optimization

**Figure 10:**
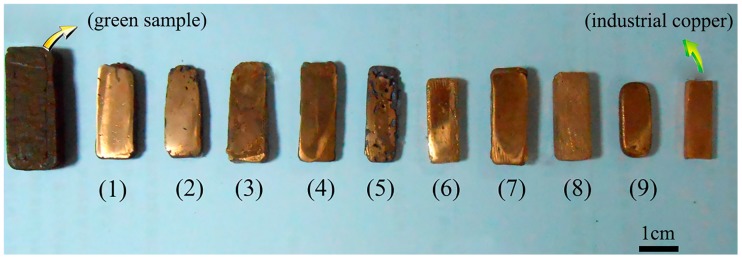
Sintered samples with different sintering parameters corresponding to Table 7.

The sintered samples with different mechanical properties were obtained by varying the sintering parameters. In this work, the orthogonal experimental design method was used to analyze the influence degree of the heating rate, highest temperature, and holding time on the hardness of the sintered samples. The optimum overall performance ratio was then selected. The heating rate, highest temperature, and holding time were determined as three experimental factors of orthogonal tests, and each factor consisted of three levels. Any two factors might have not interacted with each other. The orthogonal design table L_9_ (3^4^) was used, and the test program is presented in Table 7. The influence factors on the hardness of the sample can be described as follows: highest temperature > holding time > heating rate. The influence induced by the highest temperature on the experiments is extremely remarkable. In a certain sintering temperature range, as the temperature increases, the ability of atom diffusion would increase, and the densification sintering process was accelerated. Meanwhile, the property of parts varies significantly with the increase of temperature. As the sintering temperature increases, the grain size increases and the porosity decreases, resulting in a significant increase in the density, strength, and hardness. Meanwhile, insufficient holding time results in the strength of parts being decreased, or non-uniformity of the structure at the center and edge of the sintering boat. As the holding time increases, the density, strength, and grain size of the final products also increase. However, the effect of holding time is weaker than sintering temperature. Moreover, the density increases rapidly just in the initial stage of heat preservation. Long heat preservation times may induce extreme growth of the grain size, and result in poor performance in the properties of the products. Among the three factors, the heating rate presented the weakest effect on the hardness. Figure 10 shows the sintered samples corresponding to the data in Table 7. The orthogonal test showed that the optimal combination was heating rate (3 °C/min), highest temperature (1083 °C), and holding time (3 h). Figure 2d shows the surface of the sintered part prepared at 1350 °C with a heating rate of 3 °C/min and preserved for 3 h.

### 3.6. The Sintered Samples

**Figure 11:**
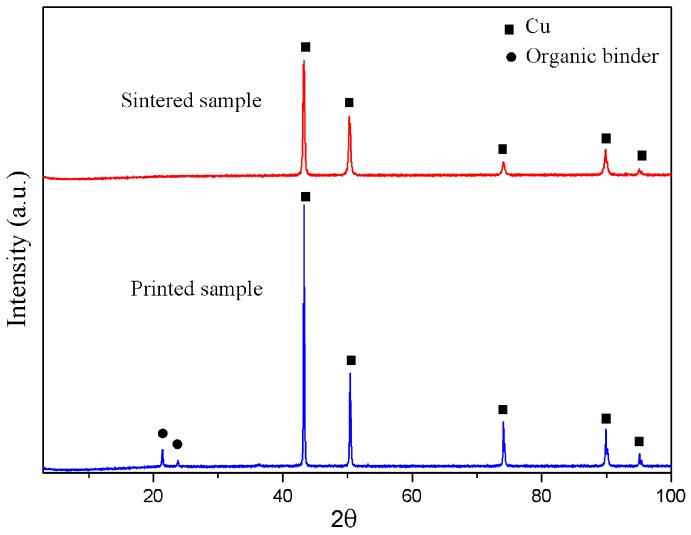
XRD patterns of the printed sample and the sintered sample with heating rate (3 °C/min), highest temperature (1083 °C), and holding time (3 h).

Good quality parts were obtained using the optimized sintering and debinding parameters. Figure 11 shows the X-ray diffraction pattern of the samples before and after debinding and sintering. XRD analysis indicates that the main component in the sintered samples was copper. After sintering, the samples show obvious shrinkage. To investigate the shrinkage of the sintered 3D components, the sample size before and after debinding and sintering was measured. Table 8 shows the amount of shrinkage in three dimensions. It can be seen that the shrinkage of the samples is almost the same in three dimensions, which indicates that the feedstock has a good uniformity.

The typical properties of copper prepared by different processes are listed in Table 9. From this point of view, it is possible for extrusion-based 3D metal to produce copper parts utilizing the PW–LDPE–SA binder system in practical applications.

**Figure 12:**
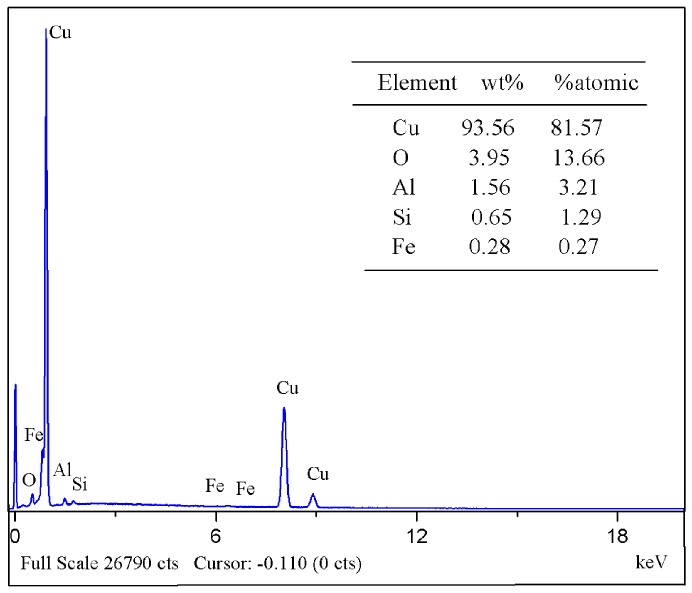
Energy dispersive X-ray (EDX) analysis of the sintered sample with heating rate (3 °C/min), highest temperature (1083 °C), and holding time (3 h).

**Figure 13:**
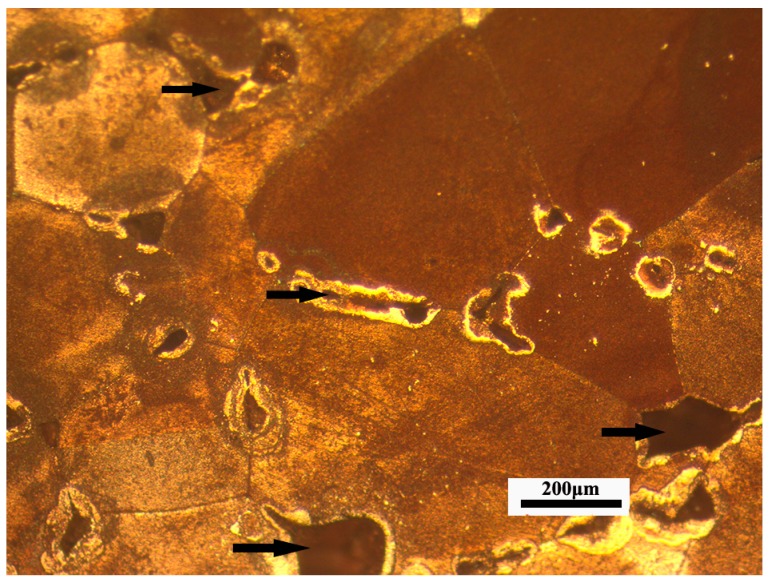
Optical Metallographic Microstructure of a sintered specimen with heating rate (3 °C/min), highest temperature (1083 °C), holding time (3 h); ×50 magnification (Black arrows indicate some microscopic defects).

The porosity of sintered parts is 8.5%. Figure 12 shows that the weight content of copper is 93.56% by energy dispersive X-ray analyses (EDX) on the surface of the sintered specimen, and the optical metallographic microstructure of a sintered specimen is shown in Figure 13. The sintered sample has a homogeneous microstructure, but some defects still exist and uniformly disperse in the sintered sample. These tiny defects are the main factors which affect the performance of the sintered specimen. These microspores can be removed by increasing the solvent degreasing.

The minimum feature size and dimensional accuracy of the parts manufactured by the proposed process are inferior to those made by laser additive manufacturing (LAM) and electron beam melting (EBM) technology. However, the extrusion-based printing process presents some advantages over traditional metal AM methods. First, traditional metal AM methods can only print specific materials. By contrast, the proposed process broadens the range of the available materials, including metals, ceramics, and composites. Second, the costs of the traditional printing equipment and materials are rather high because a high-energy beam and a device for spreading powder are required. However, the price of the self-developed printing equipment is relative low, and the raw materials are cheap and easy to obtain. Third, the efficiency of printing has been greatly improved. Traditional metal 3D printers have to use metal powders, but the proposed process uses metal paste which simplifies the process of material solidification. Furthermore, the extrusion-based printing process has extra advantages beyond the above mentioned. Notably, it has the potential to fabricate functional graded materials (FGM) and local component control (LCC) [32,33,34] parts with complex structure by using two or more dispensing heads and controlling the respective feeding rate. Fabrication of FGM and LCC parts via an additive manufacturing process is considered to be a new basic science research area [35]. The proposed process has the ability to fabricate FGM metal alloys with spatially tunable material composition.

## 4. Conclusions

We have demonstrated that metallic green samples can be produced through an extrusion-based printing process with the thermoplastic organic binder system. This article lays the foundation not only for expanding the variety of metals that can be additively manufactured, but also other particle-based materials. The following conclusions can be drawn:

(1) The raw materials with Cu particle content of 65 vol % can be prepared with the PW–LDPE–SA thermoplastic binder systems. The powder particles are homogeneously dispersed in the raw materials, and the rheological behaviour is fit for printing.
(2) During the printing process, the infill degree exerted the strongest effect on the ultimate tensile strength of the green sample, followed by the raster angle, and the layer thickness is the weakest. The orthogonal test showed that the optimal combination was infill degree (80%), layer thickness (2 mm), and raster angle (+45°/−45°). Testing also showed that the ultimate tensile strength can reach 6.73 ± 0.6 MPa.
(3) During the sintering process, the influence factors on the hardness of the sample can be described as follows: highest temperature > holding time > heating rate. The orthogonal test showed that the optimal combination was heating rate (3 °C/min), highest temperature (1083 °C), and holding time (3 h).

## Figures and Tables

**Table 1:** Composition of the raw materials.

Raw Materials		Content vol %
Cu powders		65
Organic binder	74 wt % paraffin wax (PW)	35
	23 wt % low-density polyethylene (LDPE)	
	3 wt % stearic acid (SA)	

**Table 2:** The evaluated parameters of the printing process.

Raster Angle/(°)	Layer Thickness (mm)	Infill Degree (%)
0/90	1.6	80
45/−45	1.8	70
60/−30	2.0	60

**Table 3:** The evaluated parameters of the sintering process.

Heating Rate (°C/min)	Highest Temperatures (°C)	Holding Time (h)
3	950	1
5	1000	2
8	1083	3

**Table 4:** Characteristics of Cu powders used in this study.

Powder Type	Mean Size (μm)			Specific Surface Area (m^2^/g)	Bulk Average Size (μm)
	D_10_	D_50_	D_90_		
Cu	11.96	35.57	80.98	0.19	41.06

**Table 5:** Printing conditions in this study.

Printing Conditions	Value
Extrusion temperature	160 °C
Extrusion head speed	360 mm/min
Piston speed	0.047 mm/s
Nozzle diameter	2 mm

**Table 6:** Orthogonal table of the printing parameters.

NO.	Raster Angle (°)	Layer Thickness (mm)	Infill Degree (%)	Ultimate Tensile Strength (MPa)
1	0/90	1.6	80	5.08 ± 0.45
2	0/90	1.8	70	5.57 ± 0.61
3	0/90	2.0	60	4.21 ± 0.29
4	45/−45	1.6	70	5.66 ± 0.51
5	45/−45	1.8	60	3.35 ± 0.28
6	45/−45	2.0	80	6.73 ± 0.87
7	60/−30	1.6	60	5.17 ± 0.49
8	60/−30	1.8	80	5.13 ± 0.44
9	60/−30	2.0	70	5.12 ± 0.38
$\bar{y}$_j1_	4.95	5.30	5.65	$\sum_{i=1}^{9}y_{i}=46.02$
$\bar{y}$_j2_	5.24	4.68	5.45	
$\bar{y}$_j3_	5.14	5.35	4.24	
R_J_	0.29	0.67	1.41	
Primary and secondary factor	Infill degree > Layer thickness > Orientation			
Optimal combination	Orientation (45°/−45°)—Layer thickness (2 mm)—Infill degree (80%)			

**Table 7:** Orthogonal table of the sintering parameters and hardness of the sintered samples.

NO.	Heating Rate (°C/min)	Highest Temperature (°C)	Holding Time (h)	Vickers Hardness (HV)
1	3	950	1	10.42 ± 0.87
2	3	1000	2	14.97 ± 1.02
3	3	1083	3	63.04 ± 0.60
4	5	950	3	16.12 ± 0.91
5	5	1000	1	14.85 ± 1.34
6	5	1083	2	54.65 ± 2.92
7	8	950	2	12.12 ± 1.55
8	8	1000	3	21.72 ± 1.45
9	8	1083	1	52.98 ± 4.60
$\bar{y}$_j1_	29.48	12.89	26.08	$\sum_{i=1}^{9}y_{i}=257.55$
$\bar{y}$_j2_	28.54	17.18	27.25	
$\bar{y}$_j3_	28.94	56.89	33.62	
R_J_	0.94	44	7.54	
Primary and secondary factors	Highest temperature > Holding time > Heating rate			
Optimal combination	Heating rate (3 °C/min)—Highest temperature (1083 °C)—Holding time (3 h)			

**Table 8:** Dimensional shrinkage of specimens.

Dimension	Shrinkage %
Length	20.85
Width	21.19
Thickness	21.06

**Table 9:** Properties of copper samples prepared by different processes.

Process	Vickers Hardness (HV)	Density (g·cm^−3^)	Tensile Strength (MPa)	Yield Strength (MPa)	Electric Conductivity (Ω·mm^2^/m)
Extrusion-based 3D metal printing	63.04	8.15	175	51	0.114940
Wrought Copper [28]	57	8.90	_	69	0.016903
Laser additive manufacturing (LAM) [29]	73	_	_	_	_
Electron beam melting (EBM) [28,30,31]	88	8.84	_	76	0.017774
